# GC–MS analysis of 4-hydroxyproline: elevated proline hydroxylation in metformin-associated lactic acidosis and metformin-treated Becker muscular dystrophy patients

**DOI:** 10.1007/s00726-024-03383-9

**Published:** 2024-03-10

**Authors:** Svetlana Baskal, Rene A. Posma, Alexander Bollenbach, Willem Dieperink, Stephan J. L. Bakker, Maarten W. Nijsten, Daan J. Touw, Dimitrios Tsikas

**Affiliations:** 1 Institute of Toxicology, Core Unit Proteomics, Hannover Medical School, Carl-Neuberg-Strasse 1, 30625 Hannover, Germany; 2 Department of Critical Care, University of Groningen, University Medical Center Groningen, Groningen, The Netherlands; 3 Division of Nephrology, Department of Internal Medicine, University of Groningen, University Medical Center Groningen, Groningen, The Netherlands; 4 Department of Clinical Pharmacy and Pharmacology, University of Groningen, University Medical Center Groningen, Groningen, The Netherlands

**Keywords:** Amino acids, BMD, Dialysis, GC–MS, 4-Hydroxyproline, Intoxication, Kidney injury, MALA, Mass spectrometry, PTM, Renal replacement therapy (RTR)

## Abstract

Metformin (*N*,*N*-dimethylbiguanide), an inhibitor of gluconeogenesis and insulin sensitizer, is widely used for the treatment of type 2 diabetes. In some patients with renal insufficiency, metformin can accumulate and cause lactic acidosis, known as metformin-associated lactic acidosis (MALA, defined as lactate ≥ 5 mM, pH < 7.35, and metformin concentration > 38.7 µM). Here, we report on the post-translational modification (PTM) of proline (Pro) to 4-hydroxyproline (OH-Pro) in metformin-associated lactic acidosis and in metformin-treated patients with Becker muscular dystrophy (BMD). Pro and OH-Pro were measured simultaneously by gas chromatography–mass spectrometry before, during, and after renal replacement therapy in a patient admitted to the intensive care unit (ICU) because of MALA. At admission to the ICU, plasma metformin concentration was 175 µM, with a corresponding lactate concentration of 20 mM and a blood pH of 7.1. Throughout ICU admission, the Pro concentration was lower compared to healthy controls. Renal excretion of OH-Pro was initially high and decreased over time. Moreover, during the first 12 h of ICU admission, OH-Pro seems to be renally secreted while thereafter, it was reabsorbed. Our results suggest that MALA is associated with hyper-hydroxyprolinuria due to elevated PTM of Pro to OH-Pro by prolyl-hydroxylase and/or inhibition of OH-Pro metabolism in the kidneys. In BMD patients, metformin, at the therapeutic dose of 3 × 500 mg per day for 6 weeks, increased the urinary excretion of OH-Pro suggesting elevation of Pro hydroxylation to OH-Pro. Our study suggests that metformin induces specifically the expression/activity of prolyl-hydroxylase in metformin intoxication and BMD.

**Supplementary Information:**

The online version contains supplementary material available at 10.1007/s00726-024-03383-9.

## Introduction

The biguanide metformin is the most commonly prescribed oral antihyperglycemic drug to treat type 2 diabetes (Davies et al. 2022). The exact mechanisms of metformin's pharmacological actions are not fully understood. Most pleiotropic effects of metformin are attributed to its unique property of mild but specific inhibition of complex I within the mitochondrial electron transport system (Apostolova et al. 2020).

Absorption of orally administered metformin is carrier-mediated by plasma membrane monoamine transporters and organic cation transporters (OCT). Metformin is not bound to proteins. It is eliminated primarily in its unchanged form via renal secretion. In renal tubule cells, OCT-1, OCT-2, and multidrug and toxin extrusion (MATE) transporters mediate the transcellular movement of metformin (Graham et al. 2011).

Because of its mode of elimination, patients with renal insufficiency can accumulate metformin and develop metformin-associated lactic acidosis (MALA). MALA is a severe form of lactic acidosis (plasma pH < 7.35 and plasma lactate > 5 mM) among metformin users that have a toxic metformin concentration, although this toxic metformin blood concentration is not well defined (Lalau et al. 2017). A recent study linked a metformin concentration of ≥ 9.9 mg/L with lactic acidosis, while the highest level measured in controlled clinical trials for metformin approval was 5 mg/L (Bennis et al. 2020; Kajbaf et al. 2016). In the absence of other pathophysiological conditions that cause acidosis, for example in intentional metformin intoxication, the development of lactic acidosis is named metformin-induced lactic acidosis (MILA) (Lalau et al. 2017).

The pathophysiology of MALA is characterized by reduced lactate clearance due to impaired gluconeogenesis combined with systemic mitochondrial respiratory suppression without global hypoxia, known as dysoxia, ultimately causing an increase in anaerobic metabolism (Andreis et al. 2021; Neal et al. 2015; Takiyama et al. 2011; Protti et al. 2010, 2012a, b). In patients with diabetes, the incidence of MALA is estimated to range between 3 and 10 per 100,000 person-years and is associated with a mortality rate of up to 40% (Inzucchi et al. 2014). Often, patients with MALA are treated in the intensive care unit (ICU), requiring comprehensive supportive care in combination with renal replacement therapy (RRT) like intermittent hemodialysis (HD), or when not possible in circumstances of hemodynamic instability, continuous RRT (Calello et al. 2015).

Amino acid residues in proteins undergo post-translational modifications (PTM) (Tsikas 2021, 2022). Metformin use has been associated with changes in concentrations of amino acids and their metabolites (Preiss et al. 2016). Yet, there is scarce information on whether metformin toxicity affects PTM (Wu et al. 2019; Wu 2020). Proline (Pro) residues in proteins are specifically hydroxylated to (2*S*,4*R*)-4-hydroxy-proline (OH-Pro) by prolyl 4-hydroxylases (P4H, EC 1.14.11.2) (Gorres and Raines 2010). Prolyl hydroxylases require iron and ascorbate as cofactors for their oxidation activity.

In the present study, we determined the concentration of Pro and OH-Pro in plasma, dialysate, and urine samples collected at various time points during ICU admission of a patient treated with RRT for MALA. Additionally, to gain more insight into Pro metabolism, we measured Pro and OH-Pro in serum samples of Becker muscular dystrophy (BMD) patients who received oral metformin at a therapeutic dose (Hafner et al. 2016; Hanff et al. 2018). In these studies, we used a validated stable-isotope dilution gas chromatography–mass spectrometric (GC–MS) method for the quantitative measurement of Pro and OH-Pro.

## Methods

### Case report—metformin intoxication

After the onset of diarrhea and vomiting, a 70-year-old female with a body weight of 84 kg and previously normal renal function presented at the emergency department of another hospital. She took metformin 500 mg three times daily for type 2 diabetes. Besides hypertension, she did not have any relevant other comorbidities. The patient was oliguric, and laboratory tests showed acute renal failure (plasma creatinine 606 µM or 6.8 mg/dL) with hyperkalemia (5.9 mM). Neither lactate level nor metformin blood concentration was measured. Metformin was discontinued, and extensive fluid resuscitation and treatment with an oral potassium-binding resin were initiated. Two days later, she was admitted to the ICU of the referring hospital because of decreased consciousness and hypotension. Arterial blood gas analysis showed severe lactic acidosis (pH 6.81 and lactate 16.0 mM). After intubation, 300 mL of sodium bicarbonate 8.4% was infused, and norepinephrine was initiated as a vasopressor. Throughout admission to the referring hospital and, subsequently, to our hospital, there were no signs of severe inflammation or liver dysfunction.

Subsequently, the patient was transferred to our hospital to start RRT. The RRT circuit was anticoagulated first with heparin and later with citrate. At ICU admission, the blood metformin concentration was 22.6 mg/L (175 µM), with a corresponding lactate concentration of 20 mM, bicarbonate concentration of 5 mM and a blood pH of 7.1. Hemodialysis was performed for 3 h with an AK200 Ultra S dialysis apparatus (Gambro, Breda, the Netherlands) using a hollow-fiber low-flux dialyzer (Polyflux 17L, Baxter, Utrecht, The Netherlands). The average blood flow was 256 mL/min during hemodialysis; the dialysate flow rate was 600 mL/min. The dialysate temperature was kept constant at 36 °C and the average dialysate bicarbonate concentration was 35 mM.

During hemodialysis, the metformin plasma concentration rapidly decreased, and the acidosis improved with an increase of pH from 7.26 to 7.40 in approximately 2 h. Likewise, lactate decreased from 21 mM to < 2 mM within 12 h. After intermittent hemodialysis was stopped, continuous veno-venous hemodiafiltration (CVVHDF) and, subsequently, continuous veno-venous hemofiltration (CVVH) was applied using the same dialyzer with a Prismaflex System (Baxter) with a Prismaflex ST 150 filterset. Heparin was used as anticoagulation during CVVHDF, whereas citrate predilution was used during CVVH. During CVVHDF and CVVH, the blood flow rate was 190 mL/min for both modalities, the post-dilution substitution flow rate was 2150 mL/h, and the effluent flow rate was 50.6 mL/h and 39.6 mL/h per kg bodyweight, respectively.

After 12 h from ICU admission, the metformin concentration had reached a level of < 5 mg/L (< 39 µM), a concentration that is generally considered to be non-toxic (Kajbaf et al. 2016). RRT was finally discontinued 50 h after ICU admission and the patient was discharged from the ICU a day later. Six days after ICU admission, the creatinine clearance had recovered to 51 mL/min. A month after discharge, the patient was doing well with a creatinine clearance of 64 mL/min.

### Ethical approval

Written informed consent was obtained from the patient admitted with MALA to the ICU to collect residual material and publication of this case report according to the CARE guidelines (Riley et al. 2017). Ethical approval was given by the institutional review board (METc 2014–552). After performing arterial blood gas analysis using an ABL90 FLEX as part of routine clinical care, heparin-anticoagulated blood was collected from safePICO syringes (both Radiometer, Brønshøj, Denmark). Urine and dialysate were frequently collected. Subsequently, the samples were centrifuged at 1000 × g for 12 min before storage at −80 °C.

Ethical statement Ethics and health authority approvals were obtained from the local Ethics Committee (Pilotstudie zur Untersuchung der Wirksamkeit von L-Citrullin und Metformin bei Erwachsenen mit Muskeldystrophie Becker; reference No. EKBB EK17/13) and the National Swiss Drug Agency (Swissmedic: *Pilotstudie bei Muskeldystrophie Becker*, reference No. 2013DR2067, release date 30 May 2013).

### GC–MS analysis of Pro and OH-Pro in plasma, urine, and dialysate samples

Pro and OH-Pro were analyzed by GC–MS as their methyl ester pentafluoropropionic amide derivatives as described previously for amino acids (Hanff et al. 2019; Baskal et al. 2022a). Plasma, urine, and dialysate (effluent) samples (10 µL) were evaporated to dryness using a stream of nitrogen gas. The solid residues were reconstituted in 100-µL aliquots of a methanolic 2 M HCl solution and the vials were tightly sealed. Esterification was performed by heating the samples for 60 min at 80 °C. Trideutero-methyl esters of amino acids were newly prepared in situ and used as internal standards. Subsequently, *N*-pentafluoropropionylation of the methyl esters of the amino acids was performed by using a freshly prepared pentafluoropropionic anhydride (PFPA) solution in ethyl acetate (1:4, v/v) and heating the tightly sealed glass vials for 30 min at 65 °C.

The GC–MS behaviour of Pro and OH-Pro was investigated in detail. The quantitative GC–MS method was validated in plasma and urine samples at relevant added concentrations. Quality control (QC) samples were analyzed in duplicate alongside the study samples to determine the precision and accuracy of the method for Pro and OH-Pro. For this purposes, urine was collected by a healthy non-medicated volunteer and spiked with Pro and OH-Pro at concentrations of 0 µM, each 5 µM, each 10 µM, and each 20 µM. The basal concentrations in the QC urine sample were determined to be 2 µM for OH-Pro and 6.8 µM for Pro, respectively, i.e., with a Pro/OH-Pro concentration ratio of 3.4:1. Similarly, QC was performed using plasma from EDTA-anticoagulated blood spent by the same volunteer after informed consent for Pro and OH-Pro at added concentrations of each 0 µM, 75 and 15 µM, 150 µM and 30 µM, and 300 and 60 µM, respectively. The basal concentrations in plasma were determined to be 301 µM for Pro and 21.2 µM for OH-Pro, i.e., with a concentration ratio of 14.2:1.

Metformin (Baskal et al. 2022b) was measured by GC–MS in 10-µL aliquots of samples using commercially available and ^2^H_6_-labeled metformin (Sigma-Aldrich, Steinheim, Germany; declared isotopic purity of > 99 atom% ^2^H) as the internal standard. Upon evaporation to dryness, a single derivatization with PFPA in ethyl acetate (100 µL; 1:4, v/v) for 30 min at 65 °C in tightly sealed glass was performed. Subsequent steps were as reported in previous work for metformin (Baskal et al. 2022b). Metformin was also measured in all study samples by liquid chromatography–tandem mass spectrometry (LC–MS/MS) using commercially available ^2^H_6_-labeled metformin as the internal standard (Posma et al. 2020). In plasma (0–10 mg/L), urine (0–650 mg/L), and effluent (0–10 mg/L) samples, there was a high correlation after Pearson between the metformin concentrations measured by GC–MS (*y*) and those measured by LC–MS/MS (*x*): *y* = 0.3 + 1.0*x* (*r* = 0.99) for plasma, *y* = 34 + 0.7*x* (*r* = 0.98) for urine, and *y* = 0.2 + 1.4*x* (*r* = 0.99) for effluent. For simplicity, the metformin concentrations measured by GC–MS were reported in this work. Detailed information on the method comparison is reported in Fig. S1.

### GC–MS conditions

One-µL aliquots of the extracts (toluene) were injected splitless into the GC–MS apparatus, which consisted of a single quadrupole mass spectrometer model ISQ, a Trace 1210 series gas chromatograph, and an AS1310 autosampler from ThermoFisher (Dreieich, Germany). A fused-silica capillary column Optima 17 (15 m length, 0.25 mm I.D., 0.25 µm film thickness) from Macherey–Nagel (Düren, Germany) was used. For amino acids, the injector temperature was kept at 280 °C. Helium was used as the carrier gas at a constant flow rate of 1.0 mL/min. Interface and ion-source temperatures were set to 300 and 250 °C, respectively. Interface and ion-source were set to 260 and 250 °C, respectively. Electron energy was 70 eV and electron current 50 µA. Methane was used as the reactand (buffer) gas at a constant flow rate of 2.4 mL/min for negative-ion chemical ionization (NICI). Oven temperature programs were used as described elsewhere (Hanff et al. 2019). The starting oven temperature for metformin was held at 90 °C for 0.5 min and ramped to 210 °C at a rate of 15 °C/min and then to 320 °C at a rate of 35 °C/min. In quantitative analyses, the dwell time was 100 ms for each ion in the selected-ion monitoring (SIM) mode and the electron multiplier voltage was set to 1900 V.

### Calculations

Analytical precision and accuracy were determined by standard methods as described for endogenous analytes (Tsikas 2009). Precision (actually imprecision) was calculated from replicate analyses and was reported as relative standard deviation (RSD, %). Accuracy was calculated for added concentrations of the analytes considering the basal concentrations of the analytes and is reported as recovery (%). Renal excretion, fractional excretion (FE, %) and tubular reabsorption values (T, %) were calculated as described in the Supplement.

### Statistical analyses

Data analyses were performed with GraphPad Prism version 7 (GraphPad Software, San Diego, California, USA) and R version 4.2.2 (R Foundation for Statistical Computing, Vienna, Austria). Normally distributed continuous data are presented as means with standard deviation (SD). If a normal distribution could not be assumed, data are presented as median with interquartile range.

## Results

### Analysis of Pro and OH-Pro by GC–MS—characterization of the derivatives

The GC–MS method for Pro and OH-Pro involves a two-step derivatization as described previously for amino acids (Hanff et al. 2019). The first derivatization step is HCl-catalyzed methylation of the carboxylic groups of Pro and OH-Pro. The second derivatization step is the esterification of the OH group of OH-Pro and the amidation of ring amine groups of Pro and OH-Pro by PFPA.

**Fig. 1:**
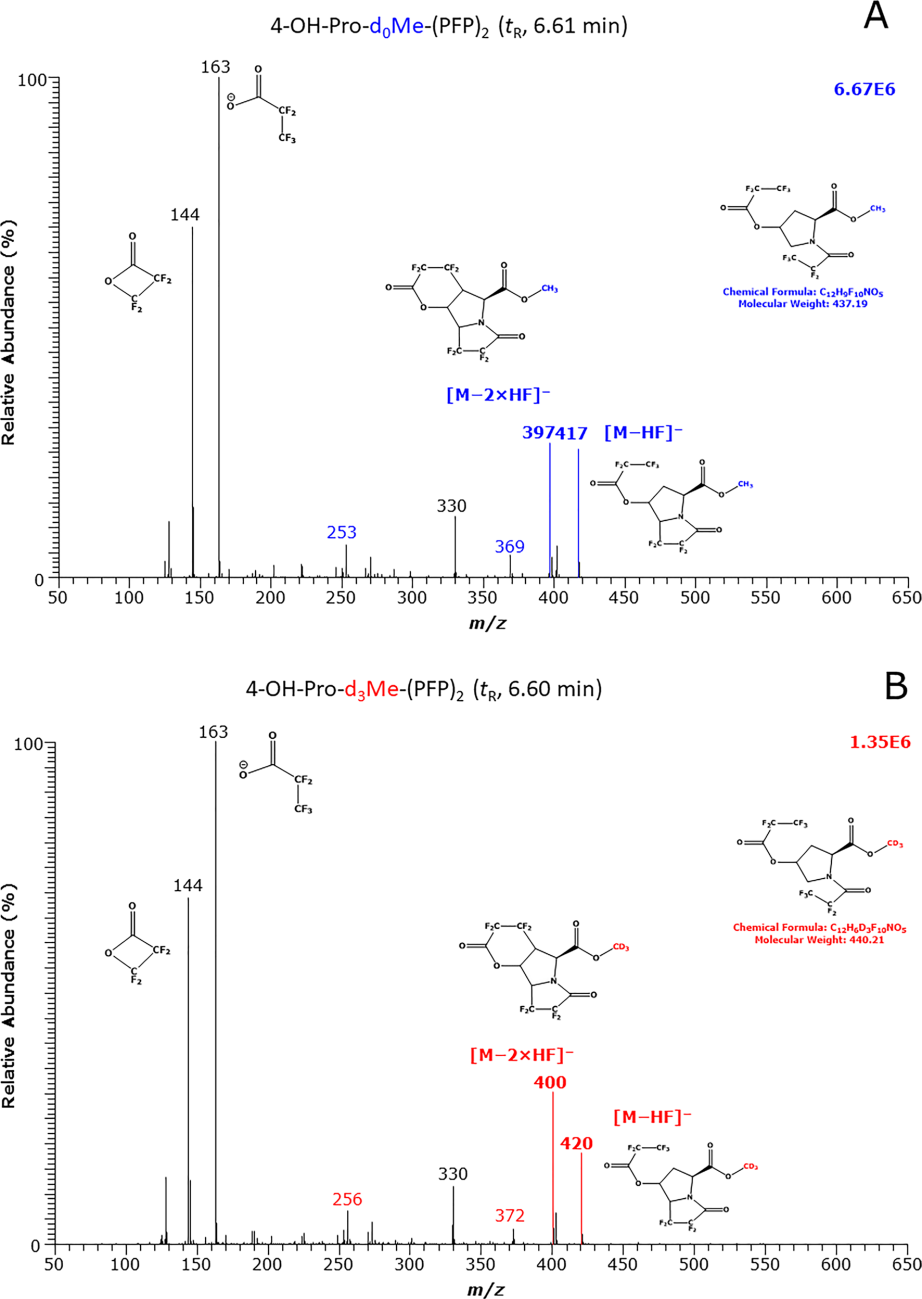

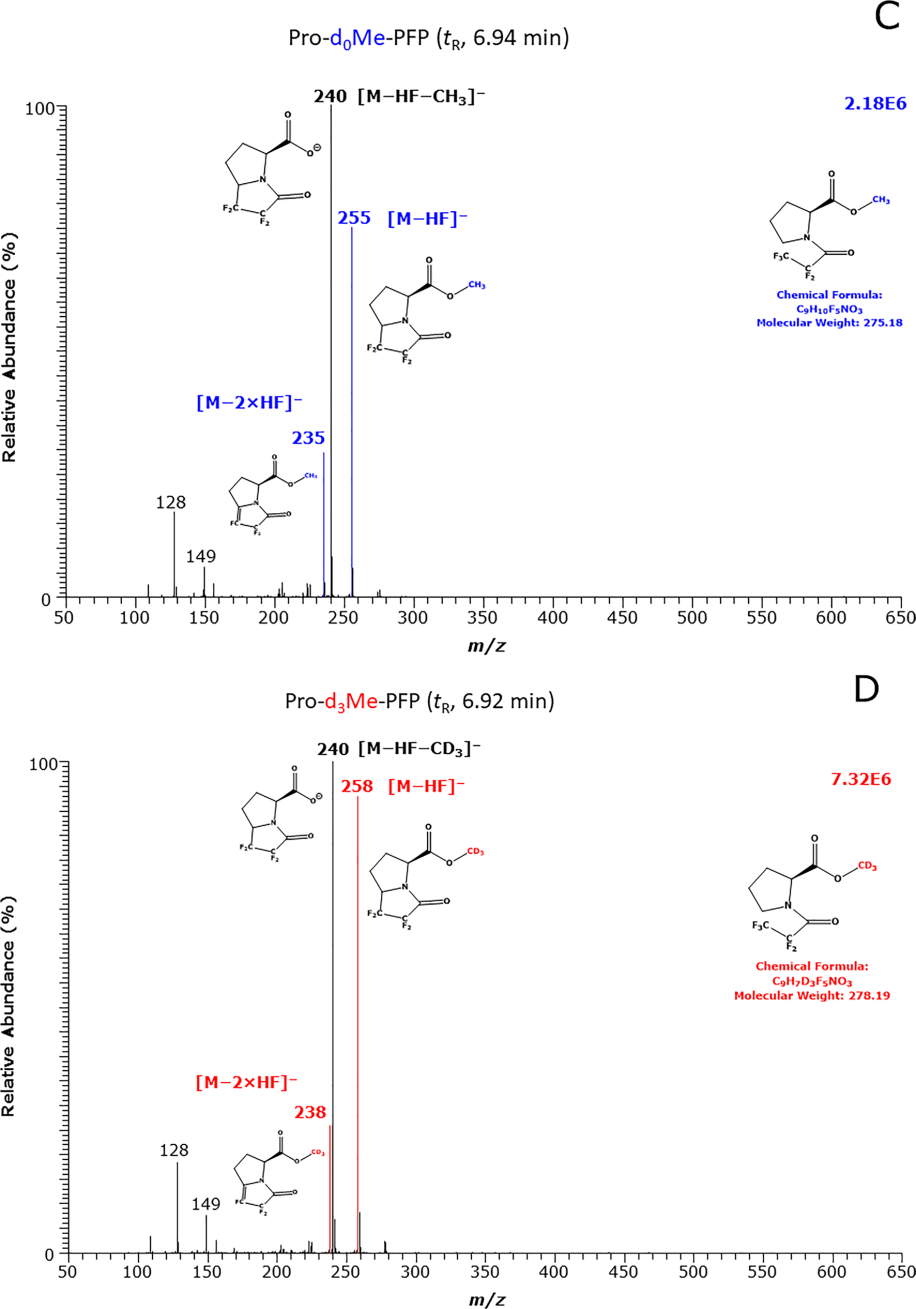
Methane negative-ion chemical ionization (NICI) GC–MS spectra obtained from the separate derivatization and analysis of **A**, **B** synthetic OH-Pro and **C**, **D** synthetic Pro (each 5 nmol injected). The methyl ester (Me) pentafluoropropionyl (PFP) derivatives were first prepared in 2 M HCl in CH_3_OH or 2 M HCl in CD_3_OD, followed by their derivatization with pentafluoropropionic anhydride in ethyl acetate. Insets indicate the proposed structures of the derivatives formed and the mass fragments produced during NICI

The GC–MS spectra of the unlabeled and deuterium-labeled methyl ester-pentafluoropropionyl (PFP) derivatives, d_0_Me-PFP and d_3_Me-PFP, of Pro (retention time, 6.94 and 6.92 min, respectively) and of OH-Pro (retention time, 6.61 and 6.62 min, respectively) are shown in Fig. 1. These mass spectra suggest that Pro forms a Pro-d_0_Me-PFP derivative and a Pro-d_3_Me-PFP derivative. Analogous, OH-Pro forms a OH-Pro-d_0_Me-(PFP)_2_ derivative and a OH-Pro-d_3_Me-(PFP)_2_ derivative. The OH-Pro derivative carries one PFP residue on the N atom and one PFP residue on the O atom on the ring position 4. The shorter retention times of OH-Pro-Me-(PFP)_2_, i.e., OH-Pro-Me-(*N*-PFP, *O*-PFP) compared to Pro-Me-PFP, i.e., Pro-Me-(*N*-PFP), suggest that the OH-Pro-Me-(PFP)_2_ derivatives are more volatile than the Pro-Me-PFP derivatives, despite its considerably higher molecular mass (437 vs. 275). This is likely due to the diester functionality of the OH-Pro-Me-(PFP)_2_ derivatives.

The most characteristic ions are mass-to-charge (*m/z*) 255 and *m/z* 235 for the Pro-d_0_Me-PFP derivative, and *m/z* 258 and *m/z* 238 for the Pro-d_3_Me-PFP derivative. The most intense ion *m/z* 240 is common to both derivatives of Pro due to neutral loss of the CH_3_ (15 Da) and CD_3_ (18 Da) groups from the methylated carboxylic groups, respectively. The most characteristic ions are *m/z* 417 and *m/z* 397 for the OH-Pro-d_0_Me-(PFP)_2_ derivative, and *m/z* 420 and *m/z* 400 for the OH-Pro-d_3_Me-(PFP)_2_ derivative. The absence of ions at *m/z* 163 and *m/z* 144 in the mass spectra of the Pro derivatives confirms the absence of an OH group in Pro (Fig. 1).

The mass spectra of the derivatives of Pro and OH-Pro differ entirely from each other. Even in the case of chromatographic coelution, Pro and OH-Pro would be able to be specifically measured in biological samples after the two-step derivatization in the order reported here. The mass spectra of Fig. 1 indicate no conversion of Pro to OH-Pro or of OH-Pro to Pro under the derivatization and GC–MS conditions used in the study. We did not observe neutral losses of 21 due to DF loss in the mass spectra of the d_3_Me derivatives, suggesting that the methyl ester group does not interact with the PFP residues of the derivatives. The abundant neutral losses of 20 due to HF strongly suggest that the PFP residues interact with the H atoms of the ring systems of Pro and OH-Pro.

### Simultaneous quantitative analysis of biological Pro and OH-Pro by GC–MS

**Fig. 2:**
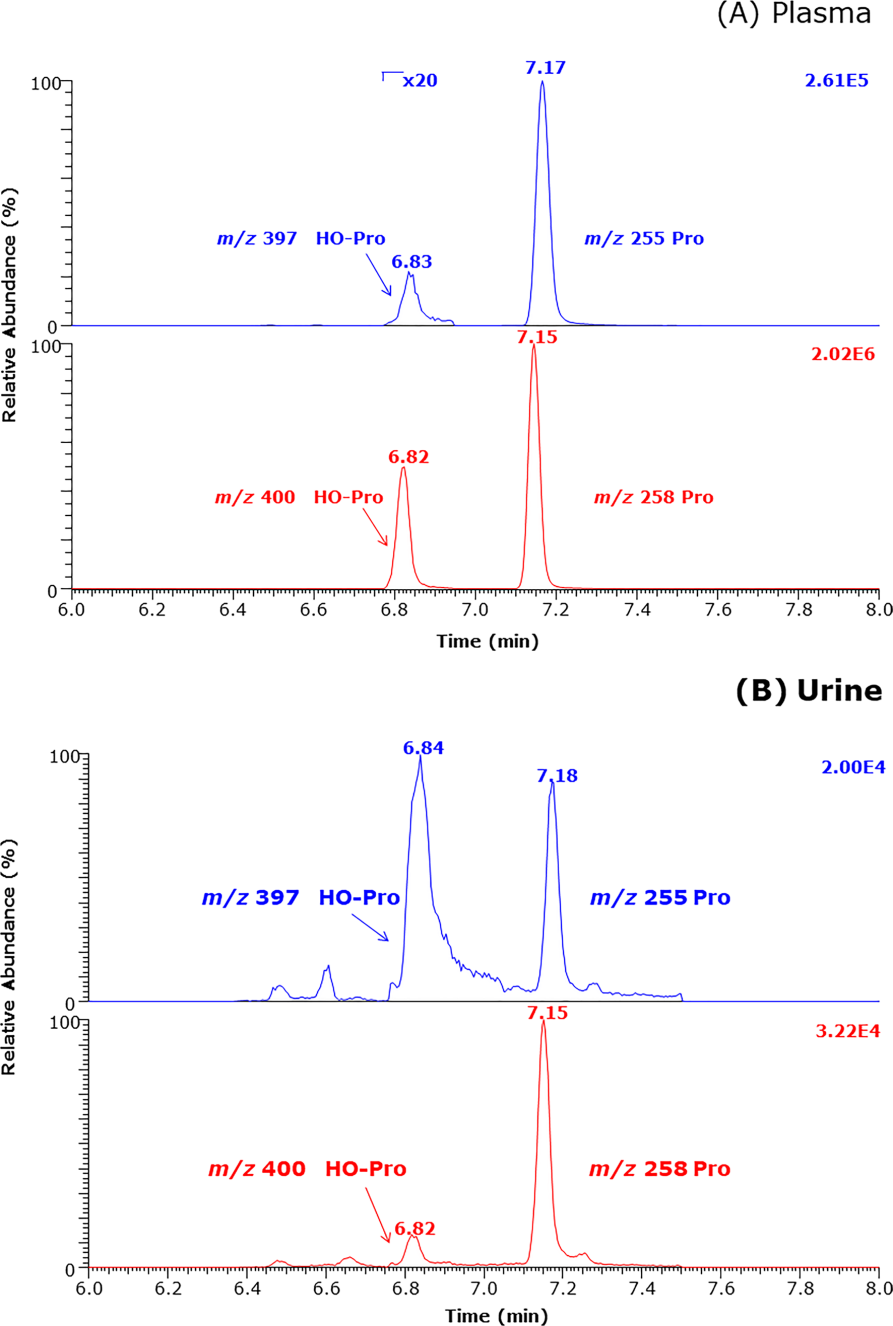
Typical partial GC–MS chromatograms from the simultaneous quantitative analysis of Pro and OH-Pro in **A** plasma and **B** urine samples of the metformin-intoxicated patient collected at about 5.5 h. SIM of *m/z* 255 and *m/z* 258 for Pro, and *m/z* 397 and *m/z* 400 for OH-Pro was performed. Upper panels show the endogenous compounds, and the lower panels show the respective internal standards. Note the magnification by a factor of 20 for the OH-Pro peak in the plasma sample. The differences in the retention times of the endogenous unlabeled and deuterium-labelled amino acids are due to the presence of deuterium in the methyl group of the internal standards

Quantitative measurements were performed in the SIM mode: *m/z* 255 and *m/z* 258 for Pro, and *m/z* 397 and *m/z* 400 for OH-Pro for the endogenous amino acids and their internal standards, respectively. GC–MS chromatograms from the simultaneous analysis of OH-Pro and Pro in a patient plasma sample and in a patient urine sample collected each at the same time point (about 5.5 h) are shown in Fig. 2.

Precision and accuracy of the GC–MS method for Pro and OH-Pro were determined using human plasma and urine QC samples. Precision (RSD, %) ranged between 0 and 11.5% in urine, and between 0.1 and 5.8% in plasma for Pro and OH-Pro, respectively. Accuracy (recovery, %) ranged between 97 and 120% in the urine, and between 116 and 120% in the plasma QC samples for Pro and OH-Pro, respectively. These data indicate an analytically satisfactory performance of the GC–MS method in human plasma and urine samples in relevant concentration ranges of Pro and OH-Pro.

We investigated potential interference between metformin (Metf) and OH-Pro in human urine samples. The peak area of unlabeled metformin (d_0_-Metf) increased linearly (*r*^2^ = 0.9949) with increasing concentration of derivatized d_0_-Metf (0–2000 µM). The peak area of deuterium-labeled metformin (d_6_-Metf) also increased linearly (*r*^2^ = 0.9331) with increasing concentration of derivatized d_6_-Metf (0–2000 µM). There was no linearity between the peak area of OH-Pro, d_3_-OH-Pro or the d_0_/d_3_-OH-Pro and the d_0_-Metf or d_6_-Metf concentration (data not shown). These observations suggest that neither d_0_-Metf nor d_6_-Metf are likely to interfere with the analysis of OH-Pro in human urine.

### Effects of renal replacement therapy—time course of metformin, Pro and OH-Pro

The concentration of metformin, creatinine (Crea), Pro and OH-Pro, and the Pro/OH-Pro molar ratio measured in plasma, urine and effluent samples during RRT are summarized in Table 1. The time course of the plasma concentrations of these analytes and their calculated renal excretion are presented in Fig. S2. The time-weighted mean plasma clearance was 85 ± 11 mL/min for CVVHDF and 56 ± 4 mL/min for CVVH. The mean time-weighted clearance of Pro and OH-Pro was 70 ± 7 and 59 ± 10 mL/min for CVVHDF, respectively, and 50 ± 5 and 38 ± 3 mL/min for CVVH, respectively (Fig. S3). Renal excretion of Pro was negligible (< 1 mL/min), whereas renal excretion for OH-Pro was highly variable.

**Table 1:** Concentrations (µM) of metformin (Metf), creatinine (Crea), proline (Pro) and hydroxyproline (OH-Pro) in plasma, effluent, and urine measured in the metformin-intoxicated patient at admission and during the treatment, and calculated Pro/OH-Pro molar ratios (Ratio)

Time (h)	5.50	7.20	9.00	10.7	11.3	12.6	15.9	30.8	35.0	40.3	46.5	48.8	50.8	53.3	57.0	62.0	63.0	64.7	66.6
Plasma (µM)																			
Metf	75.0	73.0	63.0	55.0	50.0	44.0	35.0	19.0	16.0	13.0	n.a	10.0	n.a	10.0	9.00	7.00	7.00	6.00	4.00
Crea	388	385	350	297	307	267	202	183	167	145	n.a	135	n.a	190	219	250	226	234	230
Pro	68.0	128	96.0	82.0	78.0	81.0	89.0	136	102	94.0	n.a	90.0	n.a	83.0	70.0	62.0	59.0	76.0	86.0
OH-Pro	5.94	9.24	9.22	7.9	7.74	6.52	6.58	6.77	4.52	5.53	n.a	4.15	n.a	5.51	5.15	5.77	4.51	5.51	5.58
Ratio	11.4	13.9	10.4	10.4	10.1	12.4	13.5	20.1	22.6	17	n.a	21.7	n.a	15.1	13.6	10.7	13.1	13.8	15.4
Effluent (µM)																			
Metf	1.46	1.17	1.44	1.23	1.25	2.58	3.37	3.50	2.87	4.02	5.11	3.28	2.04	1.17	0.95	0.68	0.64	0.60	0.50
Crea	2.50	1.88	2.23	2.11	2.19	4.84	6.14	4.88	4.49	6.29	6.39	5.43	3.33	2.56	2.61	3.17	3.6	3.85	4.04
Pro	11.0	8.30	10.1	8.60	8.60	29.0	43.7	81.9	51.2	90.4	111.5	75.9	37.1	23.6	27.8	17.8	18.4	13.7	15.7
OH-Pro	154.1	137.3	93.5	88.6	125.9	36.0	33.6	15.2	14.3	11.6	25.2	11.5	13.6	9.6	27.3	16.9	23.8	24.3	14.9
Ratio	0.07	0.06	0.11	0.1	0.07	0.81	1.30	5.39	3.58	7.79	4.42	6.6	2.73	2.46	1.02	1.05	0.77	0.56	1.05

Time (h)	5.00	8.80	11.0	12.0	13.0	15.3	17.6	19.5	20.8	24.3	29.1	30.5	33.4	35.0	40.3	42.6	46.5	48.9	50
Urine (µM; µM/mM)																			
Metf (µM)	28.6	80.9	69.6	60.7	54.9	45.2	37.1	32.5	29.7	23.3	20.6	18.2	16.2	15.6	12.1	11.9	10.0	9.6	18
Crea (µM)	155	344	427	384	423	415	344	256	256	188	190	286	245	267	259	252	224	220	277
Pro (µM)	36.7	103	70.2	61.4	70.4	80.6	80.9	98.7	108	113	106	106	103	87.6	68.7	88.0	82.0	73.4	110
OH-Pro (µM)	2.08	8.32	6.04	6.23	6.46	5.56	5.34	3.84	5.13	3.97	4.21	4.00	3.29	3.38	3.04	3.31	2.91	3.16	3.91
Ratio	17.6	12.4	11.6	9.90	10.9	14.5	15.1	25.7	21.1	28.5	25.2	26.5	31.3	25.9	22.6	26.6	28.2	23.2	28.1
Metf	0.58	0.62	0.65	0.58	0.57	0.53	0.55	0.72	0.64	0.64	0.80	0.6	0.61	0.46	0.36	0.21	0.18	0.16	0.12
Pro	4.40	4.40	4.50	4.10	3.90	6.00	7.10	16.8	11.4	14.4	17.4	14.0	11.1	9.20	10.7	5.60	5.10	3.60	3.90
OH-Pro	61.6	73.0	41.9	42.0	57.5	7.40	5.50	3.10	3.20	1.80	3.90	2.10	4.10	3.80	10.5	5.30	6.60	6.30	3.70

In urine, the Crea-corrected excretion rates (µM/mM) of Metf, Pro and OH-Pro are also given. n.a., not applicable

**Fig. 3:**
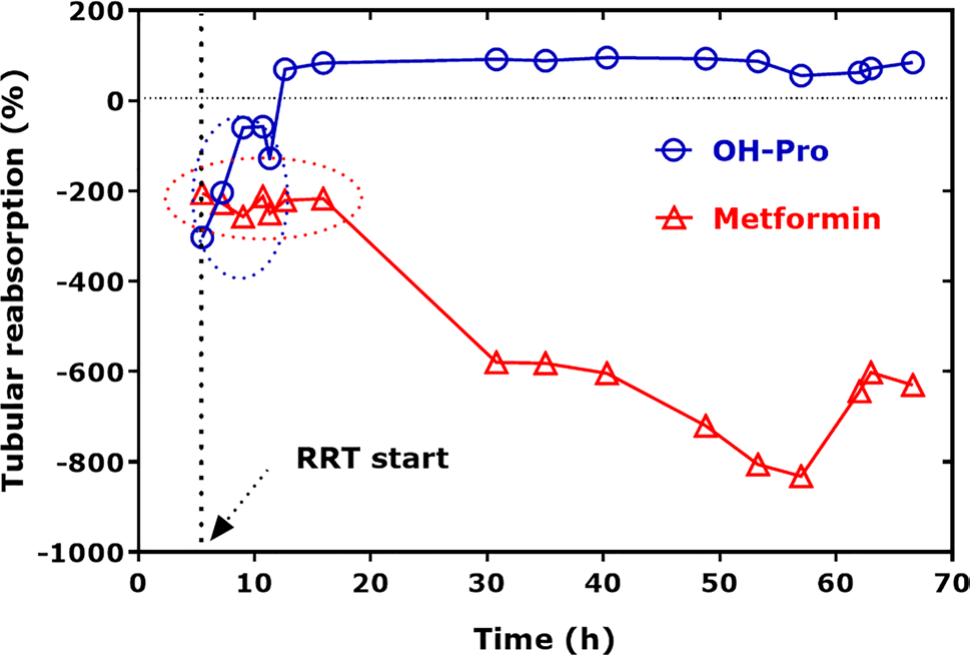
Calculated tubular reabsorption rates of metformin (triangles) and OH-Pro (circles) in the metformin-intoxicated patient throughout the renal replacement therapy (RRT; start is indicated by vertical line). Encircled are the values for metformin and OH-Pro at the first hours of the RRT

At admission, the concentration of OH-Pro and metformin were characterized by secretion of both compounds (for metformin about 200%, and OH-pro 300%, respectively) (Fig. 3). After starting RRT, tubules started to reabsorb OH-Pro gradually until reaching a value close to 100%. Metformin secretion that increased after about 15 h of ICU admission likewise indicates that tubular function was at least partially restored (Fig. 3).

During the whole admission, the plasma concentration of metformin correlated with the plasma concentration of OH-Pro (*r* = 0.762, *P* = 0.001), but not with that of Pro (*r* = 0.321, *P* = 0.208). In contrast, the urinary concentration of metformin correlated with that of Pro (*r* = 0.706, *P* = 0.001), but not with that of OH-Pro (*r* =  − 0.216, *P* = 0.619). Furthermore, the lactate concentration correlated with that of metformin (*r* = 0.8628, *P* < 0.0001) and OH-Pro (*r* = 0.5707, *P* = 0.0184), but not with that of Pro (*r* = 0.2754, *P* = 0.2824).

### Pro and OH-Pro in BMD patients and effects of metformin

In the BMD patients, the highest measured serum metformin concentration was 11 µM (Baskal et al. 2022b), which is about seven times lower than the metformin concentration measured in the plasma of the intoxicated patient at ICU admission. In the urine of the BMD patients, the highest creatinine-corrected excretion rate of metformin was determined to be 650 µM/mM (Baskal et al. 2022b). This value is comparable to the value of 580 µM/mM measured in the patient at ICU admission.

**Fig. 4:**
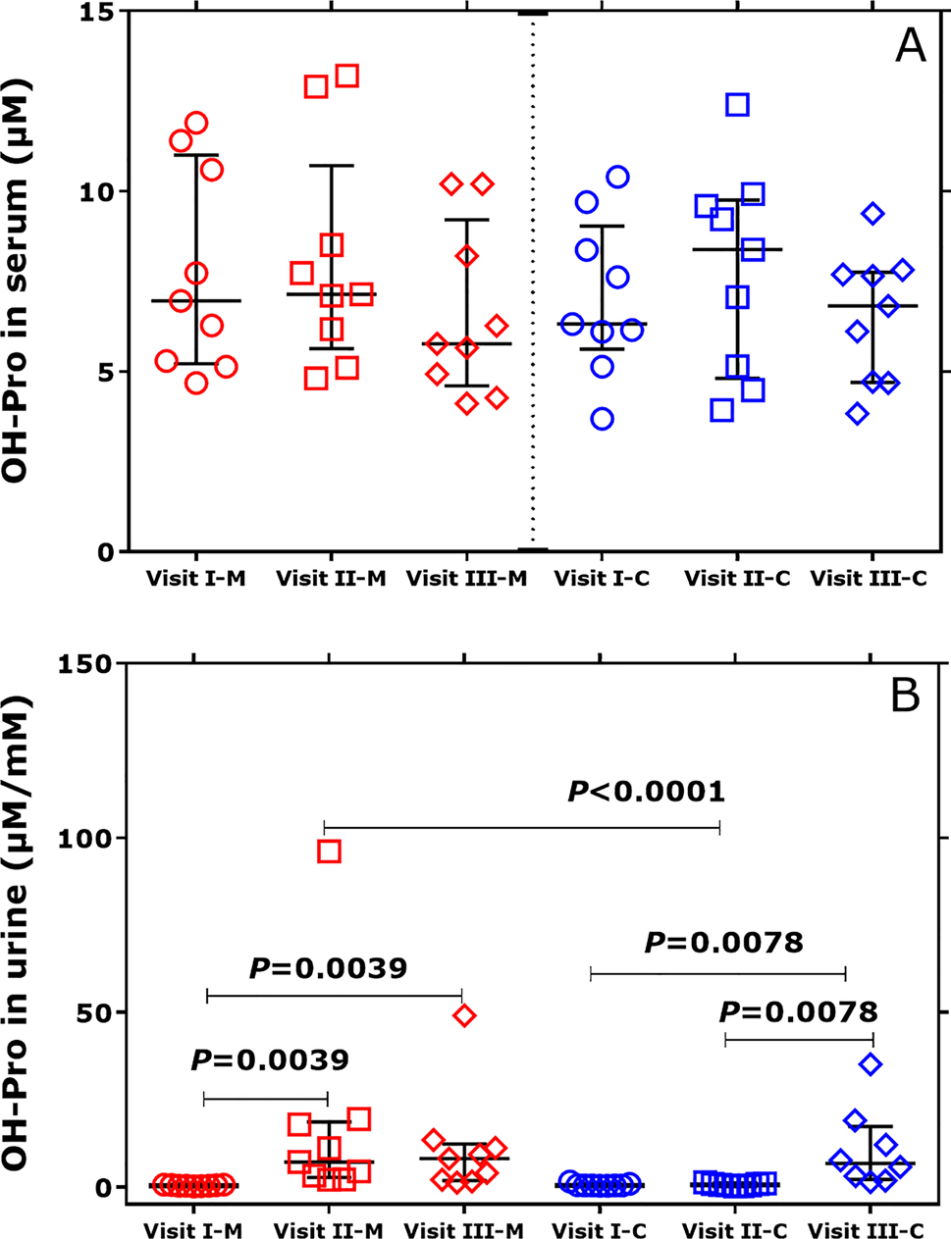
**A** Serum concentration of OH-Pro (µM) in the two groups of the BMD patients. **B** Creatinine (Crea)-corrected excretion rate of OH-Pro (µM OH-Pro/mM Crea) in the two groups of BMD patients. At Visit I, BMD patients did not receive metformin or L-citrulline. At Visit II, BMD patients received only metformin in the Metformin group (M), or only L-citrulline in the Citrulline group **C**. At Visit III, BMD patients received a combination of metformin and L-citrulline in both groups. Concentrations are given as median with interquartile range. Wilcoxon matched-pairs signed rank test within the groups and Mann Whitney test between the groups were performed. Note that there were no statistical differences between the groups with respect to the serum concentration of OH-Pro (A). See Scheme S1

In the serum and urine samples of the BMD patients collected in a previous study (Hafner et al. 2016), we additionally determined Pro and OH-Pro and calculated their molar ratio on the three visits (Scheme S1). In serum, there were no statistically significant differences for OH-Pro, Pro and the Pro/OH-Pro molar ratio between the three visits in the two study groups. The serum OH-Pro did not differ between the groups (Fig. 4A). However, the creatinine-corrected excretion rate of OH-Pro increased after single (Visit II) and combined (Visit III) administration of metformin (Fig. 4B). On Visit III, the urinary concentration of metformin correlated with that of OH-Pro (*r* = 0.762, *P* < 0.001), when taken both groups together.

## Discussion

### Effects of metformin at toxic and therapeutic doses on Pro hydroxylation

In the present work, we studied the potential effects of MALA on Pro and OH-Pro in plasma and urine samples at admission and during RRT in an ICU. Preliminary analyses indicated highly elevated OH-Pro concentrations in urine, which have hitherto not been measured in healthy and diseased humans. Therefore, we developed a stable-isotope GC–MS method that uses in situ prepared trideutero methyl esters of amino acids as internal standards after a two-step derivatization procedure (Hanff et al. 2019). We characterized structurally the derivatives of Pro and OH-Pro, and used the validated GC–MS method for their simultaneous measurement in plasma, urine and effluent samples collected at admission and subsequent long-term renal replacement therapy (RRT). Moreover, we cross-validated two orthogonal GC–MS and LC–MS/MS methods to determine metformin concentration in blood, urine, and dialysate (Baskal et al. 2022b; Posma et al. 2020).

We observed very high concentrations of OH-Pro as measured in the first urine sample taken about 5.5 h after admission to the ICU. In addition, we observed tubular secretion of OH-Pro that gradually decreased until eventually at 12 h after ICU admission nearly all of OH-Pro was reabsorbed. One may assume that toxic metformin levels or the very severe metabolic acidosis induced by metformin may interact with renal transporters for endogenous drugs and endogenous metabolites (Ivanyuk et al. 2017). This is exemplified by the result that both OH-Pro reabsorption and metformin secretion were restored to previously described levels, indicating that renal tubular function improved rather quickly over time despite the severity of acute kidney injury (AKI). AKI is a sudden decrease in kidney function that develops within 7 days (i.e., increase in serum creatinine concentration and/or decrease in urine output).

Exposure of healthy volunteers to metformin (1000 mg) was found to alter several known and unknown metabolites (Rotroff et al. 2018). In that study, OH-Pro was found to be one of the five most significantly increased metabolites in the plasma of the individuals whom received metformin (Rotroff et al. 2018). In the BMD patients of our previous study (Hanff et al. 2018), metformin alone or in combination with L-citrulline, at a therapeutic dose, did not result in appreciable changes in the serum concentrations of OH-Pro. In urine, however, metformin alone or in combination with L-citrulline increased the urinary excretion of OH-Pro. These observations suggest that the elevated OH-Pro formation observed in our intoxicated patient requires much higher metformin doses (Rotroff et al. 2018).

We measured about two times higher plasma concentrations of OH-Pro and Pro in pediatric patients (*n* = 44) under immunosuppressive therapy (tacrolimus, everolimus, cyclosporin A) due to kidney transplantation, with the mean Pro/OH-Pro molar ratio being 9.6 (Hanff et al. 2019). The mean creatinine-corrected urinary excretion rates of OH-Pro and Pro in these children were determined to be 3.2 and 11.5 µM/mM, respectively (Hanff et al. 2019). The creatinine-corrected excretion rate of OH-Pro was up to 20 times higher in the metformin-intoxicated patient of the present study for the first 12 h than under normal conditions. This comparison supports a high extent of the PTM (i.e., hydroxylation) of Pro to OH-Pro.

Besides a high extent of PTM, we cannot exclude additional concurring metabolic phenomena in our metformin-intoxicated patient, like severe metabolic acidosis on its own. Indeed, some transporters within the IMINO transport system, responsible for Pro and OH-Pro excretion, were downregulated when mice were exposed to metabolic acidosis (Moret et al. 2007). However, it is unknown whether this translates to reduced renal clearance (both metabolism and urinary excretion). Moreover, it has been reported that acidosis affects renal metformin transporter OCT-2 and MATE-1 gene expression and cellular uptake of metformin in vitro (Urakami et al. 1998; Sweet and Pritchard 1999). However, compared to controls, inducing metabolic acidosis did not affect renal clearance of metformin in rats (Gaowa et al. 2011). Metformin and creatinine are considered not to be metabolized. Among individuals with normal renal function, proline is renally extracted with a rate of 9.5 µmol/min/100 mL GFR (Tizianello et al. 1980). Whole body clearance of proline is nearly halved in patients with severe acute kidney injury due to septic or hypovolemic shock, but it is unknown to which extent the kidney contributes to the reduction in clearance (Druml et al. 1986).

There are very few reports on hyper-hydroxyprolinuria in humans (Rokkones and Loken 1968; Swarna et al. 2003). In the urine of a 6-year-old girl, the creatinine-corrected excretion rates of OH-Pro and Pro were reported as 46 mg/g creatinine (40 µM/mM) and 115 mg/g creatinine (113 µM/mM), respectively (Rokkones and Loken 1968). These values are about 1.5 and 26 times lower than the excretion rates we measured in our patient at 5.5 h, respectively. In the urine of a 2-year-old female child, the creatinine-corrected excretion rates of OH-Pro and Pro were reported as 109.6 mg/g creatinine (181 µM/mM) and 95.6 mg/g creatinine (93.9 µM/mM), respectively (Swarna et al. 2003). In both cases, hyper-hydroxyprolinuria was due to congenital renal and retinal dysplasia. These values are still about 3 and 23 times lower, respectively, than the excretion rates we measured in our patient at admission.

In normal and diabetic mice, metformin was found to accumulate for several hours in the gastro-intestinal tract as well as in the kidney (Wilcock and Bailey 1994). After uptake from the gastro-intestinal tract, the kidney is essentially the only organ that excretes metformin. Indeed, in a patient with MALA, the kidney was found to contain by far the highest metformin concentration (0.29 µmol/g), indicating accumulation of metformin in this organ (Posma et al. 2020). Of note, no samples from the gastro-intestinal tract were obtained. The renal clearance of metformin is much higher than its glomerular filtration rate indicating a considerable secretion of metformin by the proximal tubules (Somogyi et al. 1987). Structurally related drugs such as the guanidino-compound cimetidine have been shown to inhibit renal tubular secretion of metformin (Somogyi et al. 1987; Graham et al. 2011). Given the very high metformin concentration measured in the urine of our patient at 5.5 h upon admission to our ICU, it is reasonable to assume that the renal handling of endogenous metabolites including glomerular filtration and tubular secretion or reabsorption was altered by metformin, or by the severe degree of acidosis.

### Prolyl-4-hydroxylation and metformin

**Fig. 5:**
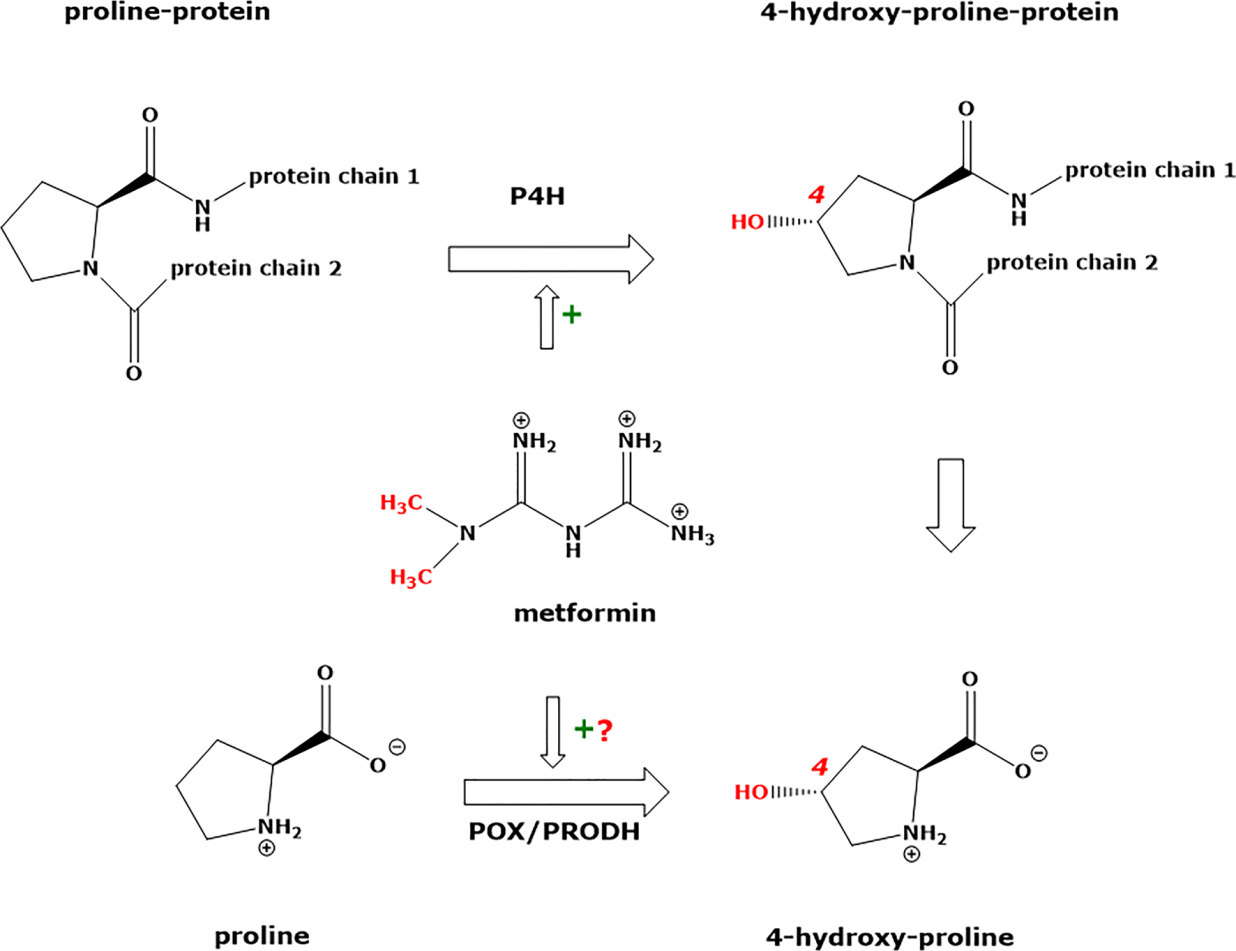
Schematic of two potential biochemical pathways of hydroxyproline (*trans*-4-hydroxy-proline [(2*S*,4*R*)-4-hydroxyproline]) formation and effects of metformin (1,1-dimethylbiguanide). P4H, prolyl-4-hydroxylase; POX, proline oxidase; PRODH, or proline dehydrogenase. The symbols + and ? mean activation and unknown, respectively

OH-Pro is the product of the PTM of Pro by prolyl-4-hydroxylase (P4H), which is a non-heme Fe^(II)^ dioxygenase. P4H is the most abundantly expressed enzyme in the proximal tubules of the kidney (Lowry et al. 1985) (Fig. 5). P4H oxidizes Pro residues in certain proteins, notably including collagen and the hypoxia-inducible factor (HIF). P4H uses α-keto-glutarate, molecular oxygen (O_2_) and ascorbate as co-substrates/cofactors (Gorres and Raines 2010). It has been shown that elevated Gln fluxes increased α-keto-glutarate concentrations, which in turn increased Pro hydroxylation in collagen (Stegen and Carmeliet 2019). In our study, the Gln/Glu concentrations in plasma and urine were normal (not shown), indicating no such effects of these amino acids.

Compared to placebo, metformin use among overweight or obese individuals with breast cancer has been associated with an increase in Pro plasma concentration (Bellerba et al. 2022). In several pulmonary fibrosis rat models, metformin use is associated with a decrease in OH-Pro concentrations in lung tissue, being attributed to AMPK activation (Wu et al. 2022).

The hypoxia-inducible factor α (HIFα) is hydroxylated on Pro residues by P4H, which allows its ubiquination and hydrolysis. Inhibition of P4H “stabilizes” the HIFα thus increasing the efflux of lactate produced by glycolysis. Inhibition of P4H also enhances gluconeogenesis from lactate in the liver, thus reducing circulating lactate levels. In mouse models of MALA and in chronic kidney disease, P4H inhibitors were found to significantly improve the survival of mice (Oyaizu-Toramaru et al. 2017). In these models, metformin was found to increase lactate blood levels (Oyaizu-Toramaru et al. 2017). Structural analogs of α-keto-glutarate are P4H inhibitors and have been reported to improve the rate of survival in experimental MALA. Succinate, the reaction product of succinyl CoA ligase (SUCLG2), is an inhibitor of P4H. As metformin inhibits SUCLG2, metformin may ultimately activate P4H, thus increasing HIFα hydroxylation to OH-Pro (Hart et al. 2019).

In our patient, we found highly elevated urinary concentrations of OH-Pro. The role of metformin in AKI is still incompletely understood. In mice, metformin aggravates AKI by inducing renal parenchymal cell death and ferroptosis (Cai et al. 2023). Metformin and its analogs can form biologically active complexes with redox-active ions including copper and iron (Lu et al. 2004; Abdelrahman et al. 2021). Metformin is also known to bind to various enzymes including cytochrome P450 and cyclooxygenase and to exert biological activity such as suppression of cancer (Guo et al. 2017; Shi et al. 2021). Although the high metformin concentrations measured in our patient may have led to depletion of iron ions by their complexation, the high OH-Pro concentrations argue against such a mechanism. Our results suggest activation of prolyl-hydroxylase by metformin (Hart et al. 2019). As metformin is eliminated via renal secretion (Graham et al. 2011), it cannot be excluded that metformin affected with OH-Pro excretion via OCT-1, OCT-2 and MATE in renal tubule cells.

The observations of the present study remain to be confirmed by analyzing plasma, urine and dialysate samples in additional cases of MALA. P4H and HIF may have a therapeutic potential in acute and chronic kidney injury (Shu et al. 2019). Prolyl-hydroxylase inhibitors are currently being investigated as potential alternative treatments for anemia in patients with chronic kidney disease (Locatelli and Vecchio 2020). Prolyl-hydroxylase inhibitors were proposed as potentially useful drugs for the treatment of lactic acidosis and MALA (Suhara et al. 2015; Oyaizu-Toramaru et al. 2017). Whether P4H inhibitors are actually useful in this context remains to be demonstrated.

Metformin can cause mitochondrial dysfunction and lactate overproduction in human platelets in vitro. Ex vivo, platelets taken from metformin-intoxicated patients have significantly lower complex I and complex IV activity compared to non-intoxicated healthy humans (Protti et al. 2012b). At therapeutical concentrations, metformin can increase sirtuin 1 (SIRT1) activity by direct binding to its NAD^+^ domain (Cuyàs et al. 2018). Such a mechanism could also apply to the inhibitory action of metformin on the mitochondrial complex I. Yet, at supra-pharmacological concentrations, metformin was found to inhibit SIRT1 activity (Cuyàs et al. 2018).

Alternative explanations of our results could be inhibition of renal proline oxidase (POX) or proline dehydrogenase (PRODH) activity by high lactate concentrations (Kowaloff et al. 1977; Summitt et al 2015; Tallarita et al. 2012; Wu 2020) and metformin-associated transporters (e.g., OCT-1, OCT-2, MATE) in renal tubule cells (Graham et al. 2011).

## Limitations

Because we only report the case of a single patient, our results should purely be regarded as hypothesis-generating. However, we did provide data with a high time resolution. As an indicator of the robustness of the observations, similar results were found among the BMD patients with normal renal function who were randomized to receive metformin.

We did neither simultaneously obtain arterial and (renal) venous blood samples, nor did we obtain inflow and outflow samples during dialysis. Therefore, calculating plasma clearance by the kidney or dialysis using the AV method was not possible. Metabolism of amino acids by the kidney should be considered when determining the renal clearance of such compounds, which is not possible within the scope of the current study. Hence, we report here only the urinary excretion of Pro and OH-Pro. It would be of great interest to determine the metabolism and excretion of different amino acids in individuals with normal renal function and in case of acute kidney injury. Moreover, creatinine, even under normal circumstances, is secreted by the proximal tubules. In the case of chronic kidney disease, the fraction of creatinine that is secreted compared to the fraction of creatinine that is filtrated by the glomerulus becomes actually even larger (Serdar et al. 2001; Garimella et al. 2021). Therefore, a fractional excretion calculation using creatinine excretion as a reference level might be not accurate due to the effects of acute kidney injury on the tubular secretion of creatinine as well. Despite that there are studies comparing the gold standard of measuring glomerular filtration with creatinine clearance, but, as far as we know, no such data have been reported on patients with acute kidney injury.

## Conclusions

GC–MS is a reliable analytical approach to measure simultaneously Pro and OH-Pro in human plasma, serum and urine samples as methyl ester-pentafluoropropionyl derivatives. Our case report suggests that metformin-induced lactic acidosis is associated with elevated concentrations of OH-Pro in urine indicating induction of renal prolyl-hydroxylase by high concentrations of metformin. Our clinical study on patients with BMD indicates that the administration of metformin increases the urinary excretion of OH-Pro. Metformin, at therapeutical doses, seems to induce specifically the expression/activity of prolyl-hydroxylase in BMD patients. Alternative routes could involve oxidation of free Pro to OH-Pro by POX or PRODH and renal OCT-1, OCT-2, and MATE transporters that mediate the transcellular movement of metformin. The underlying mechanisms warrant elucidation. Further studies on metformin intoxication are required to confirm the findings of the present study.

## Supplementary Information

Below is the link to the electronic supplementary material.

Supplementary file1 (DOCX 783 KB)

## Data Availability

Not availabe.
