# Supplementary material for: GC–MS analysis of 4-hydroxyproline: elevated proline hydroxylation in metformin-associated lactic acidosis and metformin-treated Becker muscular dystrophy patients
Source: Amino Acids. 2024 Mar 10;56:21. doi: 10.1007/s00726-024-03383-9 (PMC10925573; doi:10.1007/s00726-024-03383-9)
Supplement: Supplementary file 1 [file 726_2024_3383_MOESM1_ESM.docx]

**Supplement to**

**GC-MS analysis of 4-hydroxyproline: Elevated proline hydroxylation in metformin-associated lactic acidosis and metformin-treated Becker muscular dystrophy patients**

Svetlana Baskal,^1^ Rene A. Posma,^2^ Alexander Bollenbach,^1^ Willem Dieperink,^2^ Stephan J.L. Bakker,^3^ Maarten W. Nijsten,^2^ Daan J. Touw,^4^ Dimitrios Tsikas^1^

**Figure S1.** Comparison of metformin measurement in plasma, urine, and dialysate (effluent) using liquid-chromatography tandem-mass spectrometry (LC-MS/MS) and gas chromatography–mass spectrometry (GC-MS).

**(A. D. G)** Correlation plot of single metformin concentration values (black diamonds) in plasma, urine, and dialysate, respectively, measured with LC-MS/MS and GC-MS. Passing-Bablok regression (red line) was used to compare both measurement methods. The bias-corrected and accelerated (BCa) 95% confidence interval of this regression analysis (grey area) were generated via 1000 bootstrap samples.

**(B. E. H. C. F. I)** Bland–Altman plots showing the absolute difference (mg/L) against the average of both methods in **(B)** plasma, **(E)** urine, and **(H)** dialysate. Bland–Altman plots showing the relative difference (percentage of the average metformin concentration) against the average of both measurement methods in **(C)** plasma,**(F)** urine, and **(I)** dialysate. In all panels, the bias is represented as black dashed line, while the upper and lower 95% limits of agreement (LOA) are denoted as red dashed lines.

**Discussion of the results from method comparison**

While the difference between both methods decreased in plasma when metformin levels increased, the opposite was observed when comparing both methods in urine and dialysate. In urine, the metformin concentration was underestimated using GC-MS, while the GC-MS method overestimated the metformin concentration in dialysate.

**Figure S2.** Time course of the (A) plasma concentrations, (B) renal excretion and (C) dialysis clearance of metformin (black) and creatinine (white) during ICU admission

**Figure S3.** (A) Pro (blue) and OH-Pro (red) plasma concentrations during ICU admission. In the shaded area, the corresponding range as reported in healthy humans is displayed (Hanff et al. 2019; Wu 2020; Begou et al. 2021; Baskal et al. 2022a). (B) Renal excretion of OH-Pro; renal excretion of Pro was negligible and data are not shown. (C) Dialysis clearance of Pro and OH-Pro by intermittent hemodialysis followed by continuous renal replacement therapy.

***Becker Muscular Dystrophy patients***

Patients with Becker muscular dystrophy (BMD) received metformin 500 mg three times a day for six weeks as reported previously in detail (Hafner et al. 2016). At baseline and at the end of the treatment, serum was collected from the BMD patients to investigate the effects of metformin on the Arg/NO pathway (Hanff et al. 2018). In the present study, we newly measured by GC-MS the Pro, OH-Pro, D-5-Lys and L-5-OH-Lys concentrations in the previously collected serum and urine samples (Hafner et al. 2016).

Patients received metformin (MET group) or L-citrulline (CITR group) as the first treatments (Scheme S1). Subsequently, both groups received a combination of the drugs. The study design is illustrated in Scheme S1. Blood and urine were collected immediately prior to the first treatment (Visit I, day 0). Patients were then treated for six weeks either with 500 mg metformin (Sandoz Pharmaceuticals AG, Rotkreuz, Switzerland) thrice a day (MET Group) or with 5000 mg L-citrulline (L-citrulline drinking solution; Selectchemie Zuerich, Switzerland) thrice a day (CITR Group). At the end of this period, blood and urine samples were collected (Visit II, 6 weeks). Then, patients received both drugs at the same time, i.e., 3 × 500 mg metformin + 3 × 5 g L-citrulline for 6 weeks of treatment. Analogous, blood, and urine samples were collected immediately after the end of the combined medication (Visit III, week 12). The study was performed at the University of Basel Children’s Hospital (UKBB) as described previously in detail (Hafner et al. 2016; Hanff et al. 2018). For the present study, serum and urine samples were available from 10 patients of the MET group and 9 patients from the CITR group for the measurement of metformin (Scheme S1).

Scheme S1. Study design on the effects of metformin and L-citrulline (for six weeks) and their combination (for six weeks) in patients with Becker muscular dystrophy (BMD). For more details, see the text above.

In serum samples of untreated BMD patients (Hafner et al. 2016), we newly determined Pro and OH-Pro and calculated their molar ratio. There were no statistically significant differences for OH-Pro, Pro and the Pro/OH-Pro molar ratio between the 3 visits in the study groups (data not shown). At baseline (Visit I), median values were 8.52 µM for OH-Pro, 181 µM for Pro, and 23.9 for the Pro/OH-Pro ratio. These data and previous results (Hanff et al. 2018) suggest that BMD patients have unaltered amino acid profiles including Pro and OH-Pro. The present data also suggest that metformin (3×500 mg per day), L-citrulline (3×5000 mg per day) and their combination does not change the Pro/OH-Pro balance in the BMD patients.
